# Energy Assessment from Broiler Chicks’ Vocalization Might Help Improve Welfare and Production

**DOI:** 10.3390/ani13010015

**Published:** 2022-12-20

**Authors:** Erica Pereira, Irenilza de Alencar Nääs, André Henrique Ivale, Rodrigo Garófallo Garcia, Nilsa Duarte da Silva Lima, Danilo Florentino Pereira

**Affiliations:** 1 College of Agricultural Engineering, State University of Campinas, Campinas 13083-875, SP, Brazil; 2 Graduate Program in Production Engineering, Universidade Paulista, São Paulo 04026-002, SP, Brazil; 3 College of Agrarian Sciences, The Federal University of Grande Dourados, Dourados 79804-970, MS, Brazil; 4 Department of Animal Science, Federal University of Roraima, Boa Vista 69300-000, RR, Brazil; 5 Department of Management, Development and Technology, School of Sciences and Engineering, São Paulo State University, Tupã 17602-496, SP, Brazil

**Keywords:** animal welfare, signal analysis, acoustic communication

## Abstract

**Simple Summary:**

The objective of this study was to investigate chick vocalization through the sounds emitted during social isolation and different flock sizes. The research questions were: Which would be the ideal flock density at the first week of broiler chicken rearing? Moreover, could we verify that by using vocalization? Over 3 days, chicks (from a total of 30 birds, 1 to 3 days old) were randomly chosen and put inside a semi-anechoic chamber. Their vocalization was recorded using a unidirectional microphone connected to a digital recorder. The sound was recorded for 2 min, and the birds were removed sequentially stepwise until one bird was left inside the chamber. The fast Fourier transform was used to obtain the acoustic characteristics and the energy produced. Birds’ vocalization differed when isolated and in a group, and the energy spent in vocalizing changed depending on the size of the flock. The chicks emit a high-intensity sound when isolated (alarm call), which uses high energy. Birds spent less energy when flocked in a group and their least energy when the flock was 15 chicks in size. The signal energy also depended on the birds’ weight. The best classifier to predict the rearing flock density was the Random Forest.

**Abstract:**

Vocalization seems to be a viable source of signal for assessing broiler welfare. However, it may require an understanding of the birds’ signals, both quantitatively and qualitatively. The delivery of calls with a specific set of acoustic features must be understood to assess the broiler’s well-being. The present study aimed to analyze broiler chick vocalization through the sounds emitted during social isolation and understand what would be the flock size where the chicks present the smallest energy loss in vocalizing. The experiments were carried out during the first 3 days of growth, and during the trial, chicks received feed and water ad libitum. A total of 30 1-day-old chicks Cobb^®^ breed were acquired at a commercial hatching unit. The birds were tested from 1 to 3 days old. A semi-anechoic chamber was used to record the vocalization with a unidirectional microphone connected to a digital recorder. We placed a group of 15 randomly chosen chicks inside the chamber and recorded the peeping sound, and the assessment was conducted four times with randomly chosen birds. We recorded the vocalization for 2 min and removed the birds sequentially stepwise until only one bird was left inside the semi-anechoic chamber. Each audio signal recorded during the 40 s was chosen randomly for signal extraction and analysis. Fast Fourier transform (FFT) was used to extract the acoustic features and the energy emitted during the vocalization. Using data mining, we compared three classification models to predict the rearing condition (classes distress and normal). The results show that birds’ vocalization differed when isolated and in a group. Results also indicate that the energy spent in vocalizing varies depending on the size of the flock. When isolated, the chicks emit a high-intensity sound, “alarm call”, which uses high energy. In contrast, they spent less energy when flocked in a group, indicating good well-being when the flock was 15 chicks. The weight of birds influenced the amount of signal energy. We also found that the most effective classifier model was the Random Forest, with an accuracy of 85.71%, kappa of 0.73, and cross-entropy of 0.2.

## 1. Introduction

The study of the animal’s vocalization in terms of sound pressure level at the farm provides information on identifying distress. Vocalization studies are related to behavior, including wild animals [1,2,3], farm animals [4,5], and humans [6]. Grandin [7] describes vocalization as the active generation of sounds with specific organs, which manifest the particular state of the animal and can occur spontaneously or result from an external event. Domestic birds present various vocalizations previously recorded and described for young and adult chickens [8,9,10]. Under normal conditions, young chicks make a “peep” sound [5].

Bird vocalization is a subject broadly studied both in natural environments and in intensive housing. Vocal communication between the embryo inside the fertile egg and the hen can be carried out through vocalization, especially in the final stage of development [11]. Young chick vocalization is essential for communicating with the others in the flock [10,11] and vital to synchronize the hatching [12]. When a young chick is socially isolated from its mother, it emits a high-intensity vocalization known as an “alarm call” [10]. Previous studies have reported that the high-intensity calls emitted by isolated chicks are generated to call the hen, which emits a characteristic sound calling the chick and inhibiting the high intensity of its vocalization [10]. Some authors have highlighted that the vocalization of a chickling tends to decrease or be inhibited when there is the presence of the characteristic sound emitted by the hen [10,13,14,15]. The chick may go out looking for food due to the stress caused by social isolation, which awakens the animal’s appetite [16].

Several studies indicate the effectiveness of identifying specific individual sounds to assess the state of animal welfare [17,18,19]. This assessment method is non-invasive and can be fully automated [18]. Using alarm calls has been shown to convey a range of information about a stressful situation or a predation event, including the class of predator, level of response urgency, and the caller’s imminent behavior by using a combination of observational studies and playback experiments [16,20]. Other authors support that acoustic parameters reveal the body size of fowls and give information concerning their health and welfare condition [21].

Assessing the ideal flock density can be complicated, and high flock density might interfere with welfare status. Usually, the flock density is indicated by the breeders considering some type of scoring method indicative of negative occurrences [22] or as a result of the physiological evaluation of the animal to detect conditions such as hock burn in broilers [23]. However, stocking density usually remains an economic challenge when attending welfare norms. Technological advancement led to the use of an infrared camera, allowing it to be used as a non-invasive method for observing discomfort and risk of illness in different flock densities [24]. Among the major sources of stress that might lead to well-being deterioration are stocking density, environmental decline, inappropriate social environments, and thermal stress [25,26,27]. These aspects affect final broiler meat production due to reduced performance [28,29].

Inadequate stocking density might lead to departure from the optimal environmental rearing conditions [27]. According to Estevez [30], acoustic science should determine guidelines. However, establishing limits to stoking density based on scientific evidence may not be as easy as it appears due to the limitations needed. Moreover, limits may differ depending on the factors used to describe health and welfare, and requirements may vary for breeds. Although the recommendations for flock density vary, authors indicate that weight gain and welfare status are depreciated when stocking density decreases [25,30,31,32].

Understanding the relationship between the vocalization of animals and the environment in which they are inserted, together with studying animal behavior and its physiological parameters, allows a deep knowledge of the well-being of domestic and farm animals [33,34].

The analysis of animal vocalizations can be conducted using digital signal processing, which allows the generation of several numerical descriptions and statistical analyses [35]. According to Manteuffel et al. [36], the fast Fourier transform should be used for the digital processing and obtaining the spectra signals of digitized vocalizations. In this way, a bioacoustic analysis can be carried out. Several methods have already been developed to characterize the vocalization of animals, for example, extraction of features in the time domain, frequency domain, and cepstrum extraction, which uses the discrete Fourier transform [37].

The current study aimed to analyze the vocalization of 1-day-old male chicks exposed to a group and social isolation in different flock sizes. The research questions were: Which would be the ideal flock density at the first week of broiler chicken rearing? Moreover, could we verify that by using vocalization? We compared three classifiers to predict the chick condition using data mining. The study’s novel contribution is to analyze the chicks’ vocalization related to the energy spent during the vocalization and different group densities and develop a model to predict the well-being associated with the first week of growth group size.

## 2. Materials and Methods

A total of 30 1-day-old male chicklings (*Gallus gallus domesticus*) from the Cobb^®^ breed were purchased at a commercial hatchery. The birds were placed to rest in a closed room with the floor covered with pieces of newspaper and maintained at a room temperature recommended by the breeders. The birds had access to water and feed ration ad libitum during the trial. The group size was tested over 3 days using 1 to 3 days old chicks, and each test had 3 repetitions with randomly selected chicks.

**Figure 1:**
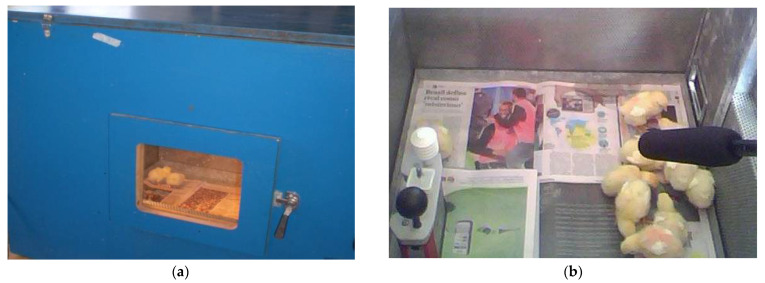
Experiment views: (**a**) Outside the anechoic chamber; (**b**) Inside the anechoic chamber showing the chickling on the floor covered with newspaper and the vocalization and the environmental temperature recording to ensure a thermoneutral ambient.

The trial consisted of randomly selecting 15 chicks from the group of 30 birds reared in another room and placing them inside a semi-anechoic chamber at a thermoneutral temperature (Figure 1a). The chamber was a partial soundproofing box [background noise of approximately 24 dB (A)] and measurements of 100 cm high × 80 cm wide × 130 cm long. The chicks were placed in the chamber for 2 min, and, after that, 1 chick was randomly selected to be removed from the group and recorded again for 2 min. We adapted the methodology adopted by Marx et al. [17] using the changes in vocalization in a stepwise decrease of group size. This procedure was repeated until one chick was left alone inside the chamber. Their vocalization was recorded using a unidirectional microphone (Yoga Ht-320a, Taiwan) placed approximately 30 cm above the birds (Figure 1b).

The microphone was connected to a digital recorder (Marantz PMD660/U3B Compact flash recorder, Japan), and the signals were digitized using a sampling frequency of 51.2 kHz. The next step was to calculate the Fourier transform [37], with 512 points, using the Hanning window, with 50% overlap, to obtain the total vocalization spectrum and extract the parameters of the signals.

The sounds were recorded in 3 repetitions for each group during data recording, and we had a total of 126 min of recording data. This setup allowed the classification of signals into three sounds: peep, short call, and alarm call. The analysis was similar to that used as a reference for speech analysis proposed by Rabiner and Juang [38]. In addition to this method, a spectral analysis of the captured signals was carried out, verifying the sound intensity in all signal frequency bands [39,40]. The Voice Editing^®^ software (ver. 2.1) was used to convert the audio signals to mp3 (MPEG Audio Layer-3 is a format for compressed audio files; 1411 kbps; 1 channel) and input the files to the computer for processing.

We calculated the energy and the centroid of the sound signals. Energy (E) is the amount of energy emitted by a sound source and is calculated using Equation (1).

(1) $$E=\int_{A}\left[\int_{0}^{∆t}I_{s}\left(r,t\right)\mathrm{dt}\right]\;\mathrm{dA}$$

where $I_{s}$ represents the acoustic intensity as a function of a point in time, and ∆t represents the time interval where the energy is measured. The spectral centroid is defined as the energy spectral mass center in each frame [41] and is calculated using Equation (2). The spectral spread quantifies how the spectrum is distributed compared to its centroid.

(2) $$\mathrm{Ce}\left(i\right)=\frac{\sum_{k=1}^{K}k.|X_{i}\left(k\right){|}^{2}}{\sum_{k=1}^{K}|X_{i}\left(k\right){|}^{2}}$$

where X_i (k) represents the components of the discrete Fourier transform of frame i, and K is half of the component spectral number used in the Fourier transform.

The spectral centroid is a good predictor of the “brightness” of a sound and is widely used in digital audio processing as an automatic measure of musical timbre. The timbre distinguishes different types of sound production, which makes a particular voice have a different sound from another [42]. We developed an algorithm in MATLAB^®^ (ver. 7.10.0) [43] that initially loads the captured audio signals. Then, after extracting the parameters, it calculates the Hilbert Transform [38] to obtain the signal’s envelope, energy, and average vocalizations. The total energy of each signal was calculated as a function of the duration of the signal in seconds [44]. The Student *t*-test with 95% significance was applied to the data to verify the energy lost in the vocalization.

The next step was to develop a classifier model using data mining. We compared three distinct classifiers kNN (k-Nearest Neighbours), Decision tree, and Random Forest, by the accuracy (Equation (3)), kappa (κ), and cross-entropy (Equation (4)). The attributes were the chick’s weight, the number of chicks, energy unit, spectral centroid, and the classes were the condition distressed or normal. The intermediate class was moved to the extremes based on the number of identified short calls [14]. We used 80% of the data to develop the model and 20% for training it.

(3) Accuracy (%)=(TP+TN)/(TP+FP+FN+TN)

where *TP* = true positives, *TN* = true negatives, *FP* = false positives, and *FN* = false negatives. We accepted that the classification was relevant when the accuracy was ≥75%.

The κ is a coefficient of dependability used to calculate two appraisers’ settlements. In the present study, we assumed that the classification was applicable when κ ≥ 0.70. The cross-entropy (H) between two probability distributions, Q from P, can be identified as H(P, Q), where P is the target distribution, and Q is the estimate of the target distribution.

(4) H(P, Q)=−∑ Qi log(Pi)

where *P*(*i*) is the probability of the event *i* in P and *Q(i)* is the probability of event *i* in Q. H ≤ 0.2 means acceptable chances for the model fitting.

**Figure 2:**
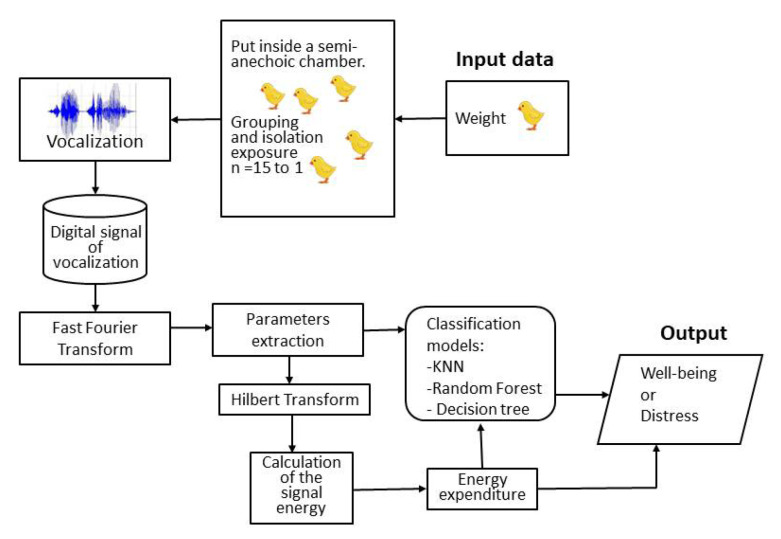
The schematic view of the vocalization signal processing.

As adopted by Marx et al. [17], we calculated the attributes in every half minute and 3 repetitions of 2 min for every tested group. However, from the 126 min of recording data, we could recover only 72 min; therefore, the total number of instances was 72. The data processing was conducted using the Rapidminer^®^ Studio (ver. 9.9), a Java-based open-source software version 9.2 (RapidMiner, Inc., Boston, MA, USA). Figure 2 shows the schematic of the data recording and analysis.

## 3. Results

### 3.1. Vocalization Output

**Figure 3:**
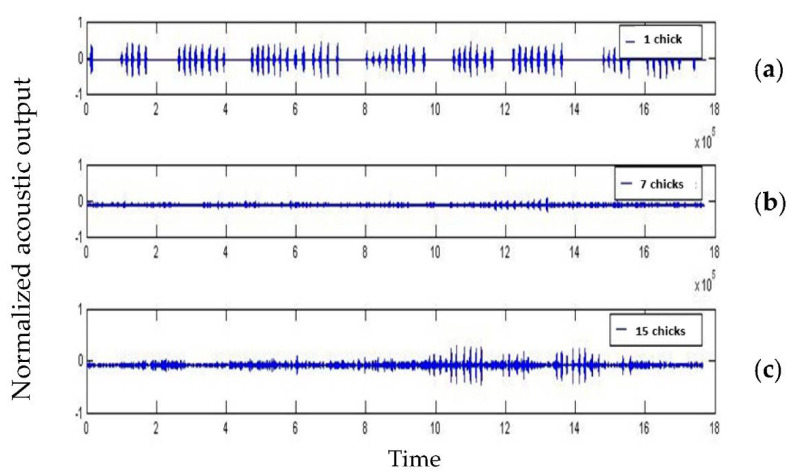
Vocalization spectral output of 1 chick (**a**) and in a group of 7 (**b**) and 15 (**c**) chicks in the time domain. 0 = baseline; normalized acoustic output varies from −1 to 1).

Since not all recordings resulted in consistent data, we present the 15, 10, 7, 5, 3, and 1 chick groups. Normalization places data points within the range proportionally to the minimum and maximum of the range. Therefore, after normalizing the distribution, the results showed three ranges of values that could be divided into three ranges: (1) the extremes (the group with 15 broiler chicks and just 1 chick), and (2) the average range (the group with 3, 5, 7, and 10 chicks). Figure 3 presents the sound’s spectrogram in the time domain equivalent to the extreme limits (1 and 15 chicks; Figure 3a,c) and the average range values (Figure 3b) inside the semi-anechoic chamber.

The vocal cords need more energy to initiate vibration for the bird to vocalize. The initial energy to start vocalization is higher than usual and tends to balance as the condition returns to normal. However, if the cause that triggered the animal’s vocalization is persistent, the energy spending tends to be potentialized, as the birds will vocalize for longer [43]. The birds’ weights did not vary (Table 1). The energy unit output (kcal/kg) was distributed with 2 extreme limits of social reaction (when there was only 1 chick-distress and 15 chicks-normal, *p* ≤ 0.05), while the other group of chicks (3, 5, 7, and 10 chicks-intermediate) remained similar. Results also showed that energy expenditure in vocalizing depended on the weight of the birds. The mean value of energy unit expended when the bird vocalized was lower when in groups of 3, 5, and 15 broiler chicks (0.036 kcal), being different with 7 and 10 chicks (0.054 kcal) and significantly higher when in social isolation (0.669 kcal).

Table 2 shows the differences in spectrograms in the instances of peep, short call, and alarm call sound for different groups of broiler chickens.

### 3.2. Classifiers Performance

We found that the kNN and Random Forest classifiers achieved the highest average prediction accuracy and kappa on the data set. However, the only cross-entropy result acceptable was given by the Random Forest algorithm (Table 3).

**Figure 4:**
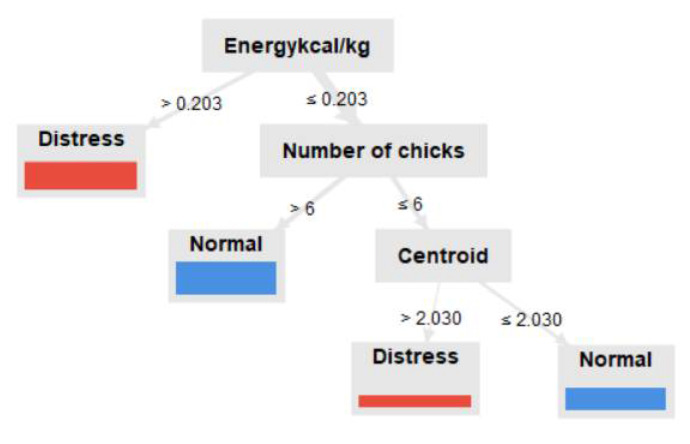
Tree-like model to predict broiler chick well-being based on data on vocalization and number of birds in the group.

We selected a tree (Figure 4) to predict the broiler’s well-being based on the elected attributes: the chick’s weight, number of chicks, energy unit, and centroid. Not all attributes were relevant to the classification of trees, and only those attributes that were helpful to the classification were considered. The main (root) attribute was the energy unit (kcal/kg). The “if-then” rules are: if the energy unit >0.203, then the bird is distressed. If the energy unit ≤0.203, then the number of chicks must be checked. If the number of chicks is >6, then the group is in normal well-being. If the number of chicks is ≤6, then the centroid must be checked. If the centroid is >2.03, then the chicks are distressed. If the spectral centroid is ≤2.03, then the chicks are distressed.

## 4. Discussion

Chicken calls are sufficiently loud to spread the alarm without being so loud as to reach the predator [1,2]. This behavior of the calling bird shows an effort to camouflage if there is any other bird nearby. However, if the distressing condition extends, the energy expenditure tends to rise due to the increased length of time spent vocalizing. Similar findings were characterized by [17] in research on social isolation in broilers. The broiler chicks tend to emit a vocalization known as an “alarm call” when subjected to stressful situations with an encoding of urgency, especially related to danger [1,43]. Marler and Evans [5] found significantly greater production of “alarm calls” in the presence of conspecific companions (when a male caller was alone or close to a female), and such behavior could affect management when rearing sexed flocks. When the power unit of energy was normalized (Table 1), the proportion of energy expenditure in terms of weight was unusually small when the birds were in groups of 15 (0.05 kcal/kg), increasing in the intermediate group (0.12 to 0.15 kcal/kg) and getting exceptionally high when isolated (14.87 kcal/kg). These values suggest that the birds were in social comfort when clustered in a group of 15 birds. At this young age, the chicks prefer to stay in groups to minimize the variation in temperature during brooding [24,25].

Results of the signal through the time domain for the vocalizations recorded indicate a different reaction to the number of chicks in the group (Figure 3), especially in the group limits tested (1 and 15), as previously expected [1,17]. Spectrographic classification using animals’ vocalization might determine the specific communication code [10]. When we classified the frequency of the calls, we observed that the chicks often vocalized peeps in the groups of 3, 5, 7, and 10 chicks, while the short call was noted when the group was 3, 5, 10, and 15 chicks. The alarm call was only seen when the chick was alone. The authors indicate that such behavior suggests an effort to camouflage if any other bird is nearby. In the present study, the “short call” had significantly less energy as predicted by current literature [45]; however, it was frequently found in the groups of 3, 5, 10, and 15 chicks.

Birds might also use “alarm calls” to provide information about the degree of danger or urgency, including heat distress [46]. We identified an association between isolation and poor well-being conditions in the trial, agreeing with previous studies [4,44]. Domestic chicks are reared by their mothers in natura and are brooded under their wings, and the physical contact and the increase in temperature are beneficial to them [25]. This concept is validated by Moura et al. [18] regarding chicks reared in thermal comfort vocalizing less than those under thermal stress. Herborn et al. [44] found that the lower distress calls on the first days of rearing, the higher the average broiler weight and less cumulative mortality at the slaughter age. Therefore, besides the energy loss in the first days, the economic impact increases when the whole cycle is considered.

The energy unit was the most important attribute, followed by the number of chicks in the group (flock density) and the vocalization spectral centroid. The model indicates that a group of more than six broiler chicks apparently lead to normal well-being condition and relates to the vocal spectral centroid characteristic of less than 2.03 to normal. Previous studies [45,46] found high centroid values correlated to distress calls, and in the current study, we only found this correlation for centroid values to be superior to 2.03. A previous study [47] suggests that young broiler chicks were less fearful under high flock-density conditions than in low-density conditions. Associating the information from the classifier model and the normalized mean of a 1-day-old chick’s weight and energy expenditure, we observed that the ideal group of young chicks was within the range of 6 and 15 birds.

Broiler vocalization is a helpful tool [48,49] and, associated with machine learning, might provide the needed input for the automated assessment of broiler welfare [50]. Further studies are required in this field at different stages of growth since the bird’s mass increases over time, and heat transfer between the broilers also modifies during the growth period, as well as the interactions and behavior. The present study did not consider environmental issues other than the lack of heat that might impact animal welfare, such as the relative humidity inside the chamber and the animal handling during the experiment. Those variables might affect animal welfare to some extent.

## 5. Conclusions

The young chick’s energy expenditure indicated high distress during isolation. The model result suggested that a group of 6 broiler chickens and higher until 15 birds might be ideal if the vocal spectral centroid is less than or equal to 2.03. We found that the Random Forest presented the best model for the three tested classifiers to predict the chick’s well-being. We also noticed that the chicks’ energy spent during the vocalization differed in various group densities.

Although the study utilized a relatively small number of birds compared to commercial flocks, it presents new information regarding the vocalization response of broiler chickens in specific flock densities.

## Figures and Tables

**Table 1:** The normalized mean of a 1-day-old chick’s weight and energy expenditure during vocalization in a group and alone.

Number of Chiacks	Mean Weight (kg/Bird)	Mean Standard Error (kg/Bird)	Energy Unit (kcal/kg)	Reaction	Mean Standard Error (kcal/kg)	Spectral Centroid
1	0.045 a	0.002	14.87 c	Distress	12.501	1.24
3	0.047 a	0.002	0.16 b	Intermediate	0.118	2.73
5	0.046 a	0.000	0.14 b		0.068	2.37
7	0.048 a	0.001	0.14 b		0.122	1.30
10	0.046 a	0.000	0.12 b		0.088	1.74
15	0.047 a	0.001	0.05 a	Normal	0.029	1.96

The values with the same letters do not differ (*p* ≥ 0.05).

**Table 2:** The example of the spectrogram and occurrence of peep, short call, and alarm call sound during the experiment related to the group of broiler chickens.

Spectrogram *	Vocal Reaction	Occurrence
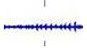	Peep-Intermediate	Often in groups of 3, 5, 7, and 10 chicks.
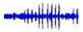	Short call-Normal	Occasionally in the group of 3, 5, 10, and 15 chicks.
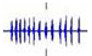	Alarm call-Distress	Continuously, when there was just 1 chick.

* A spectrogram is an image of the signal spectrum frequencies as it varies with time.

**Table 3:** The comparison results of the tested classifiers.

Classifier	Accuracy (%)	Kappa	Cross-Entropy
kNN	85.71	0.72	0.3 *
Random Forest	85.61	0.73	0.2
Decision tree	71.43	0.46 *	0.6 *

* = inadequate values.

## Data Availability

Data will be available from the corresponding author upon request.
